# Atypical features of hepatic veno-occlusive disease/sinusoidal obstruction syndrome after inotuzumab ozogamicin in adult patients with acute lymphoblastic leukemia

**DOI:** 10.1007/s44313-025-00077-3

**Published:** 2025-04-22

**Authors:** Kyung-Hun Sung, Daehun Kwag, Gi June Min, Sung-Soo Park, Silvia Park, Sung-Eun Lee, Byung-Sik Cho, Ki-Seong Eom, Yoo-Jin Kim, Hee-Je Kim, Chang-Ki Min, Seok-Goo Cho, Seok Lee, Jae-Ho Yoon

**Affiliations:** 1 Department of Hematology, Catholic Hematology Hospital and Leukemia Research Institute, Seoul St. Mary’s Hospital, College of Medicine, The Catholic University of Korea, 222 Banpodaero, Seocho-Gu, Seoul, 06591 Korea; 2 Hematology, Department of Internal Medicine, Mokdong Hospital, College of Medicine, Ewha Womans University, Seoul, Republic of Korea

**Keywords:** Inotuzumab ozogamicin, Hepatic veno-occlusive disease, Acute lymphoblastic leukemia

## Abstract

**Purpose:**

Inotuzumab ozogamicin (INO) has demonstrated a safe bridging role to allogeneic hematopoietic stem cell transplantation (HSCT) in patients with relapsed or refractory B-cell acute lymphoblastic leukemia (R/R B-ALL). However, hepatic veno-occlusive disease/sinusoidal obstruction syndrome (VOD/SOS) is frequently observed. This study aimed to identify significant features of INO-associated VOD/SOS.

**Methods:**

We reviewed seven cases of hepatic VOD/SOS that developed either during INO salvage or after allogeneic HSCT following INO-induced complete remission (CR). Diagnosis and severity grading of VOD/SOS were based on the revised criteria from the European Society for Blood and Marrow Transplantation. Defibrotide was used to treat severe to very severe cases.

**Results:**

Four patients developed VOD/SOS during INO salvage therapy (at 21 and 36 days post-INO1, 77 days post-INO3, and 21 days post-INO5), while three were diagnosed at 2, 5, and 10 days post-HSCT following INO-induced CR. Doppler ultrasonography revealed preserved portal vein flow (range 10.2–26.0 cm/sec) and normal hepatic artery resistive index (RI, range 0.56–0.74) in all but one patient (RI 0.83). Despite this, all patients presented with massive ascites and progressively elevated total bilirubin levels. All cases were classified as severe to very severe; six were treated with defibrotide and one underwent liver transplantation. Most patients ultimately died owing to VOD/SOS progression.

**Conclusion:**

Post-INO VOD/SOS manifested as two different clinical settings and was characterized by preserved portal vein flow, which complicated diagnosis. Despite timely defibrotide administration, clinical outcomes were poor. These findings emphasize the need for vigilance and potential consideration of prophylactic strategies for prevention of INO-associated VOD/SOS.

## Main body

Hepatic veno-occlusive disease (VOD), also known as sinusoidal obstruction syndrome (SOS), is considered one of the most life-threatening complications following hematopoietic stem cell transplantation (HSCT) [1]. Inotuzumab ozogamicin (INO), an anti-CD22 monoclonal antibody–drug conjugate (ADC), has been shown to improve remission rates and survival in patients with relapsed/refractory acute lymphoblastic leukemia (R/R ALL). However, INO is also associated with an increased risk of hepatic VOD/SOS [2]. Diagnosing VOD/SOS can be challenging owing to similar differential diagnoses and overlapping hepatic conditions [3]. We previously reported a case of atypical VOD/SOS following INO salvage treatment that led to a delayed diagnosis [4]. This study investigated additional cases of INO-associated VOD/SOS with atypical features.

Among 80 patients who received INO salvage therapy at Seoul St. Mary’s Hospital between January 2019 and December 2023, seven (8.7%) were diagnosed with VOD/SOS during INO salvage treatment (*n* = 4) or after a second allogeneic HSCT following INO-induced remission (*n* = 3). VOD/SOS stage and grade were classified according to the revised criteria from the European Society for Blood and Marrow Transplantation (EBMT) [3]. Additional data, including laboratory findings, Doppler ultrasonography, CT imaging, and clinical complications, were collected retrospectively from medical records.

The clinical courses of INO treatment and the development of VOD/SOS in the seven patients are shown in Table 1. The median patient age was 41 years (range 27–66 years). Four patients (Number #1–4) developed VOD/SOS during INO salvage therapy, with a median onset of 144 days (range 26–264) after the first INO dose. This corresponded to 8–21 months post-initial allogeneic HSCT. All four had grade 2 hepatic GVHD at the time of INO administration and VOD/SOS diagnosis. Pre-INO liver imaging conducted owing to prior hepatic GVHD suspicion showed normal or mildly abnormal findings, including preserved portal vein flow (19.7 cm/s in patient #1 and 26.7 cm/s in patient #3). Notably, despite the clinical progression to VOD/SOS, Doppler ultrasonography continued to show preserved portal vein flow (e.g., 21.3 cm/s in patient #1). In contrast, three patients developed VOD/SOS shortly after allogeneic HSCT following INO-induced remission (on days 2, 5, and 8 post-transplant). Five patients received two or fewer cycles of INO to minimize toxicity for the preparation of allogeneic HSCT, while two (Cases #2 and #3), who were ineligible for allogeneic HSCT, received more than three cycles. All cases were classified as severe VOD/SOS per the refined EBMT criteria [3]. Doppler ultrasonography performed to assess hemodynamic instability showed unexpectedly well-preserved portal vein (PV) flow higher than 10 cm/s and hepatic artery resistance index (RI) less than 0.75, considering the amount of ascites and the severity of VOD/SOS. Hepatic artery RI higher than 0.8 and peak systolic velocity (PSV) higher than 100 cm/sec were observed in only one patient (Number #1). Liver biopsies were performed in three patients (Numbers #1, #2, and #6), all of whom showed typical histopathological findings of sinusoidal dilatation and extravasation of red blood cells. Defibrotide was initiated on the day of diagnosis in six patients. One patient (Case #1) underwent liver transplantation instead. Only one patient recovered fully after 30 days of defibrotide therapy. The remaining six died of VOD/SOS progression, with complications including multi-organ dysfunction (*n* = 2) and sepsis (*n* = 3).

**Table 1:** Patient characteristics and outcome measures

**No**	**Sex/Age**	**Disease status at INO**	**Cycles of INO**	**Onset of VOD/SOS Post-INO or HSCT**	**Severe Grade**	**Doppler ultrasonographic findings**	**Histopathologic findings**	**Treatment course**
1	F/49	Post-HSCT EMR	1	Post-HSCT D + 8 M, Relapse with hepatic GVHD **INO salvage D + 36**	Very severe	**Portal vein flow: 21.3 cm/s** RI: 0.83, PSV: 107.6 cm/s Spleen: 9.0 cm Ascites: Large amount	Sinusoidal dilatation with congestion and foamy histiocytic infiltration. Moderate cholestasis. Presence of bile duct dystrophy	VOD progression, DDLT Pneumonia sepsis **Dead** (Post-INO 5.5mo)
2	M/35	Post-HSCT EMR + BMR	5	Post-HSCT D + 16 M, Relapse with hepatic GVHD **INO salvage D + 250**	Very severe	**Portal vein flow: 17.5 cm/s** RI: 0.56, PSV: 35.8 cm/s Spleen: 6.0 cm Ascites: Large amount	Diffuse sinusoidal dilation and focal hemorrhage with minimal cholestasis	Defibrotide VOD progression, Sepsis **Dead** (Post-INO 8.6mo)
3	M/66	Post-HSCT EMR	3	Post-HSCT D + 21 M, Relapse with hepatic GVHD **INO salvage D + 264**	Very Severe	**Portal vein flow: 11.2 cm/s** RI: 0.62, PSV: 47.4 cm/s Spleen: 7.3 cm Ascites: Large amount	NA	Defibrotide VOD progression **Dead** (Post-INO 11mo)
4	M/45	Post-HSCT EMR + BMR	1	Post-HSCT D + 8 M, Relapse with hepatic GVHD **INO salvage D + 26**	Very severe	**Portal vein flow: 10.2 cm/s** RI: 0.74, PSV: 22.8 cm/s Spleen: 10.0 cm Ascites: Moderate amount	NA	Defibrotide VOD progression **Dead** (Post-INO 23 days)
5	M/41	Post-HSCT BMR	2	INO salvage D + 93 **Post-HSCT D + 5**	Very Severe	**Portal vein flow: 26 cm/s** RI: 0.72, PSV: 88.6 cm/s Spleen: 13.3 cm Ascites: Moderate amount	NA	Defibrotide VOD progression, sepsis **Dead** (Post-INO 4.7mo)
6	M/27	Post-HSCT BMR	1	INO salvage D + 43 **Post-HSCT D + 8**	Very Severe	**Portal vein flow: 10.9 cm/s** RI: 0.59, PSV: 59.3 cm/s, Spleen: 14.2 cm Ascites: Moderate amount	No lobular and no porto-periportal activity. No fibrosis. Mild cholestasis. Sinusoidal dilation and hepatocyte dropout	Defibrotide VOD progression, sepsis **Dead** (Post-INO 2.9mo)
7	F/36	Post-HSCT EMR + BMR	1	INO salvage D + 99 **Post-HSCT D + 2**	Very Severe	**Portal vein flow: 19.9 cm/s** RI: 0.74, PSV: 90.7 cm/s, Spleen: 10.1 cm Ascites: moderate amount	NA	Defibrotide **Alive** (Post-INO 7mo)

*INO* Inotuzumab ozogamicin, *VOD/SOS* Veno-occlusive disease/ sinusoidal obstruction syndrome, *HSCT* Hematopoietic cell transplantation, *DDLT* Deceased donor liver transplantation, *EMR* Extramedullary relapse, *BMR* Bone marrow relapse, *RI* Resistive Index, *PSV* Peak Systolic Velocity

## Discussion

Hepatic VOD/SOS is clinically characterized by jaundice with hyperbilirubinemia, weight gain, and painful hepatomegaly resulting from sinusoidal endothelial damage. While typically observed post-allogeneic HSCT, diagnosis remains complex [5]. Although liver biopsy is a diagnostic standard, it carries a high risk of bleeding. Therefore, imaging techniques such as Doppler ultrasound have been introduced, which provide insight into hepatic hemodynamic instability, with findings such as PV flow < 10 cm/s, hepatic artery RI > 0.75, and PSV > 100 cm/s considered suggestive of disease progression, as reduction of PV flow and change into hepato-fugal flow are observed in patients with severe grade VOD/SOS [6, 7].

**Fig. 1:**
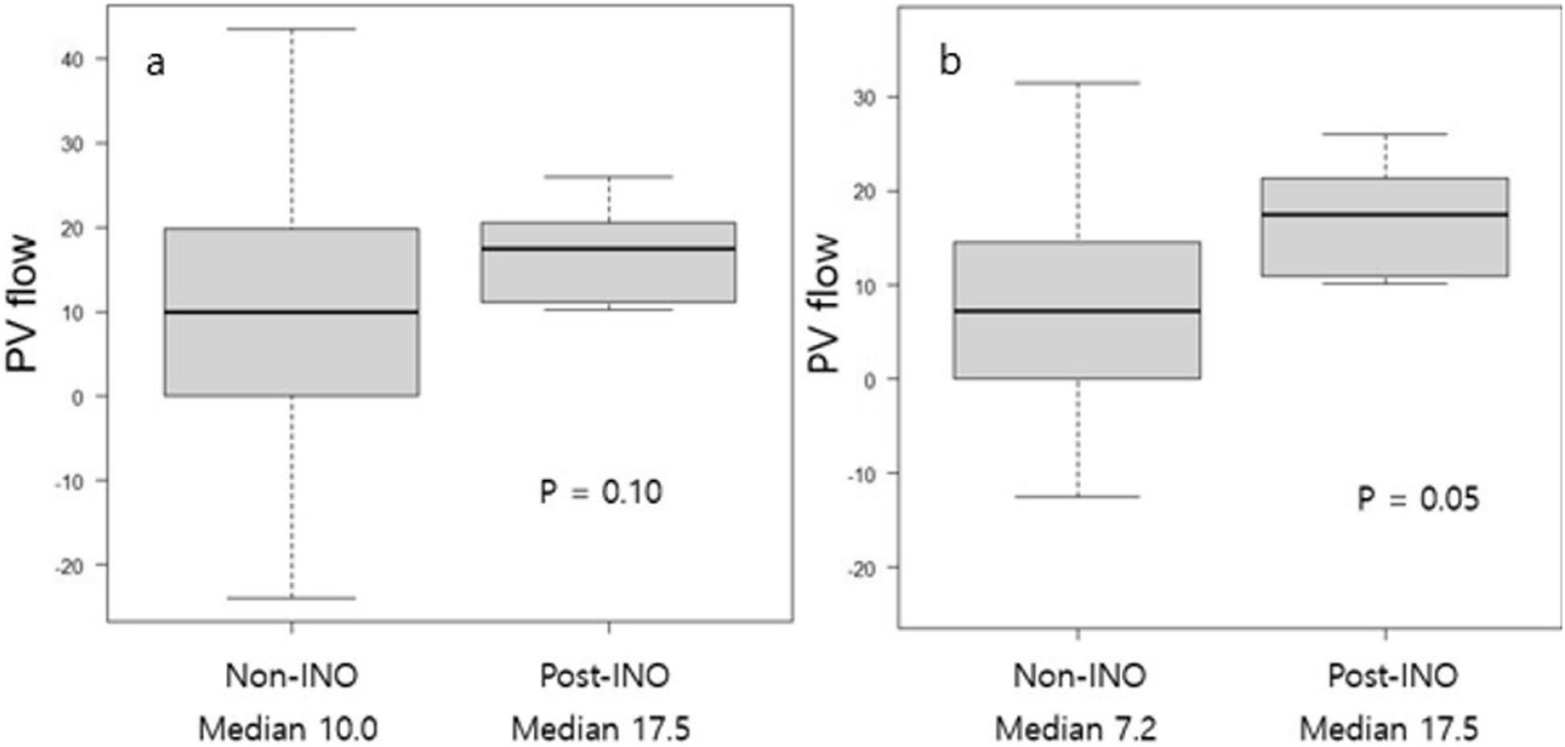
Portal vein flow compared with post-INO and non-INO according to SOS/VOD grade. **a** severe to very severe grade. **b** very severe grade. INO, inotuzumab ozogamicin; PV, portal vein

In our experience, INO-related VOD/SOS presented in two clinical contexts: after allogeneic HSCT following INO salvage and the other during INO treatment (often after post-HSCT relapse). All cases were classified as very severe, but Doppler findings (PV flow, hepatic RI, and PSV) remained unexpectedly preserved. As hemodynamic changes in VOD/SOS typically stem from sinusoidal obstruction (related to sinusoidal vessel narrowing and thrombotic occlusion), the preserved PV flow in these patients may reflect a distinct pathophysiological mechanism. Compared to traditional post-HSCT VOD/SOS,, our cases demonstrated more preserved PV flow (Fig. 1) [8]. A possible explanation is that antibody drug conjugates release cytotoxic calicheamicin intracellularly, which is excreted by the biliary system. Additionally, non-targeted conjugates may damage the liver [9], potentially via pathways, such as intrahepatic cholestasis or drug-induced liver injury rather than sinusoidal endothelial damage. This mechanism may also relate to ADC-associated thrombocytopenia [10].

Clinically, INO remains a key treatment option for patients with R/R ALL. This inevitably leads to the use of INO in patients who have previously experienced GVHD (Patients #1–4). Diagnostic challenges arise from the overlap of VOD/SOS with hepatic GVHD or toxic hepatitis. Our findings suggest that persistent bilirubin elevation despite corticosteroid treatment, in conjunction with CT evidence of hepatic sinusoidal congestion and periportal edema may favor the diagnosis of VOD/SOS over GVHD exacerbation. Moreover, the timing of clinical symptoms is a key distinguishing factor. Although ascites and weight gain are often late manifestations of other hepatic complications, they tend to appear early in VOD/SOS. Recognizing this pattern can aid in early suspicion and intervention. While Doppler ultrasonography may not detect early VOD/SOS in this context, additional modalities such as liver stiffness measurement and hepatic venous pressure gradient assessment—as recommended by the 2023 EBMT guidelines—may improve diagnostic accuracy [3]. Importantly, our findings reveal a clinicopathologic-radiologic discrepancy in INO-related VOD/SOS. In such cases, treatment should not be delayed if clinical features meet diagnostic criteria, especially for probable VOD/SOS [3].

This case series highlights that INO-related VOD/SOS presents with more preserved PV flow on Doppler imaging compared to traditional post-HSCT VOD/SOS, which were unrelated to INO, with distinct patterns depending on whether the condition develops during INO treatment or after allogeneic HSCT at INO-induced CR. The preserved PV flow and normal hepatic RI in these patients pose significant diagnostic challenges. Despite prompt defibrotide administration, outcomes were poor, with high mortality from VOD/SOS progression and multi-organ failure. These findings emphasize the need for heightened vigilance, prompt treatment, and potential prophylactic strategies in patients receiving INO. Further studies are required to elucidate the underlying mechanisms and improve management of this severe complication.

## Data Availability

No datasets were generated or analysed during the current study.
